# Critical Review of Synthesis, Toxicology and Detection of Acyclovir

**DOI:** 10.3390/molecules26216566

**Published:** 2021-10-29

**Authors:** Yan-Ping Wei, Liang-Yuan Yao, Yi-Yong Wu, Xia Liu, Li-Hong Peng, Ya-Ling Tian, Jian-Hua Ding, Kang-Hua Li, Quan-Guo He

**Affiliations:** 1 School of Life Science and Chemistry, Hunan University of Technology, Zhuzhou 412007, China; wyp2226547993@163.com (Y.-P.W.); wyy5082010@163.com (Y.-Y.W.); plhhnu@163.com (L.-H.P.); tianyaling0212@163.com (Y.-L.T.); 2 Zhuzhou People’s Hospital, Zhuzhou 412001, China; lxfz0826@163.com (X.L.); dingjianhua168168@163.com (J.-H.D.); 3 Hunan Qianjin Xiangjiang Pharmaceutical Joint Stock Co., Ltd., Zhuzhou 412001, China; yly733@163.com

**Keywords:** acyclovir, pharmacology, synthesis, toxicology, analytical methods

## Abstract

Acyclovir (ACV) is an effective and selective antiviral drug, and the study of its toxicology and the use of appropriate detection techniques to control its toxicity at safe levels are extremely important for medicine efforts and human health. This review discusses the mechanism driving ACV’s ability to inhibit viral coding, starting from its development and pharmacology. A comprehensive summary of the existing preparation methods and synthetic materials, such as 5-aminoimidazole-4-carboxamide, guanine and its derivatives, and other purine derivatives, is presented to elucidate the preparation of ACV in detail. In addition, it presents valuable analytical procedures for the toxicological studies of ACV, which are essential for human use and dosing. Analytical methods, including spectrophotometry, high performance liquid chromatography (HPLC), liquid chromatography/tandem mass spectrometry (LC-MS/MS), electrochemical sensors, molecularly imprinted polymers (MIPs), and flow injection–chemiluminescence (FI-CL) are also highlighted. A brief description of the characteristics of each of these methods is also presented. Finally, insight is provided for the development of ACV to drive further innovation of ACV in pharmaceutical applications. This review provides a comprehensive summary of the past life and future challenges of ACV.

## 1. Introduction

**Figure 1:**
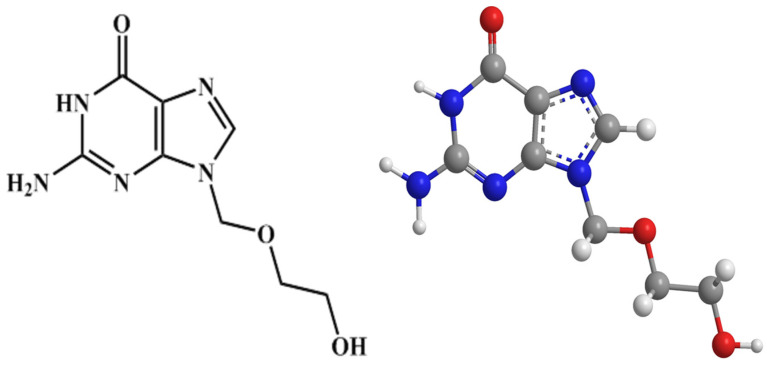
Molecular structure of ACV.

The structure of acyclovir (9-((2-hydroxyethoxy)methyl)guanine) (ACV) is illustrated in Figure 1. As an antiviral drug of guanine nucleoside analogues, ACV is one of the most commonly used antiviral drugs all around the world. It is considered the beginning of a new era of antiviral therapy, due to its high selectivity and low cytotoxicity [1]. It is used to treat herpes viruses, such as herpes simplex virus (HSV), varicella-zoster virus (VZV) and Epstein–Barr virus (EB) effectively, with little effect on normal cells [2,3]. In infected cells, it has a powerful inhibitory effect on viral DNA, preventing its synthesis. ACV is also one of the most important essential medicines for establishing essential care systems, being included in the WHO Model List of Essential Medicines (October 2013).

ACV, as an effective and selective antiviral drug, is one of the most common drugs in the worldwide pharmaceutical market. Low cost and high yield synthesis processes are essential for the development of ACV. In addition, with this extensive clinical application, it was found that ACV effectively treats herpes, but with some adverse effects, such as causing acute renal impairment. The State Drug Administration of China issued a notice on the revision of ACV formulation instructions (April 2009), mentioning that patients need to be carefully observed for signs and symptoms of renal failure when applying ACV therapy. In addition, the amount of ACV used is influenced by individual differences, with the elderly, pregnant women and children needing to use ACV with caution. Due to the above toxicological and adverse effects, it is particularly important to detect and analyze the level of ACV, and many analytical methods have been developed to effectively identify and quantify the level of ACV present not only in commercial pharmaceutical preparations, but also in human urine and serum, which has played a positive role in ensuring safe drug use on patients.

This review summarizes the discovery and pharmacology of ACV, from its ability to inhibit the process of viral coding. The development of ACV is comprehensively described from three points of view, including preparation method, surname change and dosage form, to provide a theoretical basis for the clinical efficacy of this drug. To address the toxicological studies of ACV and the challenge of how ACV can be safely administered, existing analytical methods for ACV, such as spectrophotometry, high performance liquid chromatography (HPLC), liquid chromatography/tandem mass spectrometry (LC-MS/MS), electrochemical sensors, molecularly imprinted polymers (MIPs), and flow injection–chemiluminescence (FI-CL) are reviewed. A brief description of the characteristics of each of these methods is also given separately. Finally, the paper provides hot spots of current ACV research and insights into future challenges for further innovation of ACV in pharmaceutical applications. This research work is of high theoretical significance with a good clinical utility.

## 2. The Discovery and Pharmacology of ACV

Acquired Immune Deficiency Syndrome (AIDS) spread rapidly around the world in the 1970s. Confronted with the spread of the AIDS epidemic, chemists began to look for nucleoside analogues for antiviral drugs. ACV was discovered by Schaeffer et al. [4] in 1974 through a search for inhibitors of adenosine deaminase. It entered into clinical trials due to the intense preclinical investigation in 1977. Subsequently, ACV was developed by Burroughs Wellcome (B.W., UK) as an acyclic nucleoside drug for specific anti-herpes viruses, which were first marketed in the world in 1981 [5,6]. Since then, ACV has become one of the most widely sold antiviral drugs in the world and has demonstrated its value in a variety of clinical settings. It is mainly used to control the symptoms of HSV, which causes herpes simplex; or varicella-zoster virus (VZV; a type of herpes virus), which causes shingles and chickenpox [7]. ACV is effective against HSV-1 and HSV-2, and against VZV, while being relatively weak against Epstein–Barr virus (EBV) and cytomegalovirus (CMV). Meanwhile, ACV has some effect against hepatitis B virus (HBV). Besides, with in-depth studies of ACV, ACV can also be used in combination with other agents [8,9,10]. For example, it may combine with zidovudine (AZT) to treat AIDS or with human lymphoblastoid interferon to treat chronic hepatitis B. Currently, ACV is most commonly prepared in six formulations, including tablets, capsules, injections, eye creams, eye ointments and eye drops [1]. Local application treats herpetic keratitis, herpes simplex and herpes zoster. Oral or intravenous injection can effectively treat herpes simplex encephalitis, genital herpes and immune deficiency patients with herpes simplex infection.

**Scheme 1:**
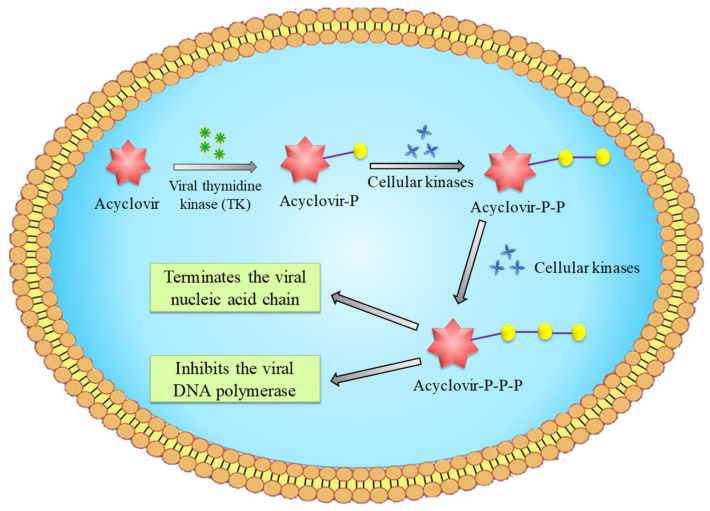
Activation of ACV in infected cells.

ACV can significantly inhibit the synthesis of viral DNA in infected cells, without affecting the DNA replication of non-infected cells. Viral replication relies on nucleotides supplied by host cells as the raw material for viral DNA or RNA synthesis. Many viruses can use viral thymidine kinase (TK) to convert RNA thymine nucleoside into monophosphate nucleoside. Subsequently, with the action of cell kinase, monophosphate nucleoside is further converted into diphosphate and triphosphate nucleoside to be used by the virus. Similarly, ACV, the antiviral nucleoside drug, as the raw material for viral replication is converted to its monophosphate derivatives by TK, which is a reaction/process that does not occur to any significant extent in uninfected cells (Scheme 1). Monophosphate is then further converted into diphosphate and active triphosphate, under the catalysis of cellular kinases. ACV triphosphate is involved in the DNA chain of the synthesizing virus. However, the uptake of this compound by the virus blocks the extension of the DNA strand. This is attributed to the lack of the 3′ -hydroxyl group, which blocks the replication of the viral nucleic acid. In addition, although ACV triphosphate competes with deoxyguanosine triphosphate (dGTP) for binding to viral DNA polymerase, the affinity of viral DNA polymerase to ACV triphosphate is much higher than that of dGTP, which results in the interference of polymerase combining with the viral replication templates or primers, thus inhibiting the activity of viral polymerase. Finally, the synthesis of viral DNA and the proliferation of viruses are blocked.

## 3. The Preparation of ACV and Its Dosage Forms

### 3.1. The Synthesis of ACV

**Scheme 2:**
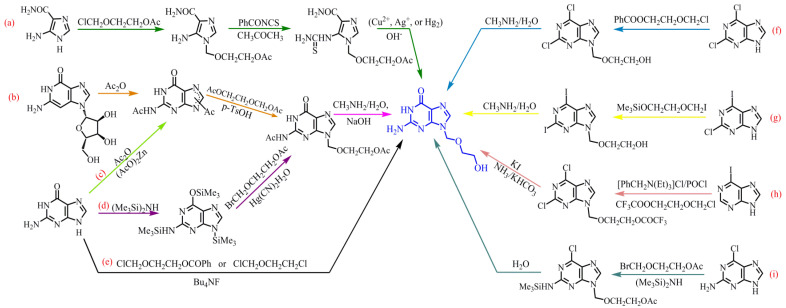
The diagram of main synthetic routes of ACV.

With the advantages of low toxicity, good tolerability, high efficiency and broad spectrum, ACV is in great demand in the market and therefore the exploration of its chemical synthesis methods has been widely reported. A great deal of literature has been reported about the synthesis methods of ACV. The main synthesis routes of ACV can be divided into the following three categories according to different raw materials, shown in Table 1. The main synthesis routes are summarized in a diagram of Scheme 2.

#### 3.1.1. Synthesis from the 5-Aminoimidazole-4-carboxamide

The synthesis of ACV starting with 5-aminoimidazole-4-carboxamide is displayed in Scheme 2a. Briefly, 5-aminoimidazole-4-carboxamide undergoes alkylations with 3-oxy-4-chlorobutanol acetate (ClCH_2_OCH_2_CH_2_OAc), after which the product is condensed with benzoyl othiocyanate (PhCONCS) by heating reflux, in acetone (CH_3_COCH_3_). Finally, ACV is obtained by cyclodesulfurization hydrolysis under alkaline aqueous solution, containing a suspension of a slight excess of the metal salt (Cu^2+^, Ag^+^, or Hg_2_) [11,12]. This scheme cleverly adopts the method of cyclodesulfurization by using heavy metal salts to make it possible for the synthesis of ACV from 5-aminoimidazole-4-carboxamide. Although the strategy has the advantages of mild reaction conditions and simple operation, the raw material is not very easy to obtain, so it would potentially take a long time to carry out the work. More importantly, it is not environmentally friendly to add the heavy salts during the production process.

#### 3.1.2. Synthesis from Guanine and Its Derivatives

As biochemical reagents, guanosine and guanine, which have extensive application, are relatively easy to obtain. In the pharmaceutical industry, they are often used for the synthesis of pharmaceutical products. In 1992, Wang et al. [13] reported a synthesis method of ACV from guanosine by three steps. This synthesis route is displayed in Scheme 2b. First, guanosine was acylated by acetic anhydride to become *N, N’*-diacetylguanine. After that, the condensation of *N, N’*-diacetylguanine and 2-oxy-1, 4-butanediol diacetate (AcOCH_2_CH_2_OCH_2_OAc) was done in the presence of *p*-totuenesulfonic acid in organic solvents. Finally, ACV was produced by aminolysis in the presence of methylamine. It is recommended as an ideal route for designing experiments, due to the accessible raw materials, simple industrial conditions and high yield.

However, there are numerous methods to synthesize ACV by using guanines as the starting material, which use fewer steps, or just one reaction step. Typically, it can be prepared by acylation of guanines followed by condensation and ammonolysis. In 1988, Matsumoto et al. [14] proposed a convenient and economical synthesis of ACV from guanine (Scheme 2c). Guanine can be acylated to N, N’-diacetylguanine by Ac_2_O. Subsequently, *N^2^*, *O*-diacetylacyclovir is prepared by the condensation of *N*, *N’*-diacetylguanine and AcOCH_2_CH_2_OCH_2_OAc in the presence of *p*-toluenesulfonic acid (*p*-TsOH) in dimethyl sulfoxide (DMSO). Finally, ACV is successfully synthesized by using ammonia–methanol (NH_3_–CH_3_HO) as an organic solvent. Considering the need to be environmentally friendly and the production yield of ACV, Chen and Wang [15] consulted and modified Matsumoto’s synthesis route to improve the ACV production yield to 52%. They used (the cheaper) toluene instead of using DMSO as the solvent for the condensation reaction, which greatly reduced the amount of reprocessing work. Meanwhile, a methylamine solution (CH_3_NH_2_–H_2_O) was employed instead of NH_3_–CH_3_OH in ammonolysis, which mitigated the application of a large number of organic media. Interestingly, differing from Matsumoto and Chen, Luo [16] prepared the ACV by the silanization of hexamethyldisiline (HMDS), then by condensation with 3-oxy-4-bromobutanol acetate (BrCH_2_OCH_2_CH_2_OAc), and finally by alkaline hydrolysis, as shown in Scheme 2d. The silanization route does not easily form 7-by-product of guanine, due to the large steric hindrance, and condensation is carried out by Hg(CN)_2_ as a catalyst, which is able to eliminate 7-by-product, thus greatly improving the purity of the product. This synthetic route has a potential industrial value, as it increases the total yield to 72.3%. The aforementioned methods of preparing ACV from guanine involve a three-step reaction but reducing and simplifying the reaction steps is also important for the synthesis process. Hakimelahi and Khalafi-Nezhad [17] took a different approach. They developed a general and rapid procedure for the preparation of ACV, which was produced by the condensation of guanine with 3-oxy-4-chloro-butanol benzoate (ClCH_2_OCH_2_CH_2_OCOPh) or chloromethylchloroethyl ether (ClCH_2_OCH_2_CH_2_Cl) in the presence of tetrabutylammonium fluoride (Bu_4_NF) in one step, achieving the condensation and deprotection of the acyl protecting group and nucleophilic displacement of the halogen atoms. This synthesis route is shown in Scheme 2e.

#### 3.1.3. Synthesis from Other Purine Derivatives

It is commonly seen that purine derivatives as starting material provide promising options for the preparation of ACV. For example, Kelley and Schaeffe [18] adopted 2, 6-dichloropurine as the raw material. After carrying out the condensation with 3-oxy-4-chloro-butanol benzoate, methylamine ammoniation and hydrolysis, ACV was finally obtained (Scheme 2f). Similarly, when 2-chloro-6-iodopurine was used as raw material [19], it needed to undergo condensation with Me_3_SiOCH_2_CH_2_OCH_2_I, ammoniation and hydrolysis to obtain ACV (Scheme 2g). Moreover, Han et al. [20] invented a method involving an efficient and selective process for the production of 9-(2-hydroxyethoxymethyl)-guanine (ACV). As Scheme 2h shows, with the presence of benzyl triethyl ammonium chloride, 3-oxy-4-chlorobutanol trifluoroacetate alkylated the 6-iodopurine. After that, it was chlorinated with phosphorus oxychloride and iodized with potassium iodide. Finally, it underwent alkaline hydrolysis by potassium bicarbonate to successfully produce the ACV. In addition, Scheme 2i presents a synthesis method starting with 2-amino-6-chloropurine. After silanization with hexamethyldisilane, condensation with 3-oxy-4-bromobutanol acetate and hydrolysis, ACV was finally produced [21].

### 3.2. Structures Modification for Improving ACV Performances

Generally, the synthesized ACV is a white crystalline powder with poor water solubility, fat solubility, oral and external absorption, and low bioavailability. Therefore, it is crucial to modify the ACV to be lipophilic, to achieve better properties. For instance, the palmitoyl group is introduced into ACV molecules [22,23] to prepare a lipophilic prodrug of ACV palmitate (ACV-C16). This prodrug can be prepared into liposome gel for skin use, which effectively improves the transdermal delivery and bioavailability of ACV. ACV and succinic anhydride were synthesized as 9-(2- single acidated butanedioic ethoxymethyl) guanine in basic conditions with pyridine as a solvent [24]. The water solubility of ACV was increased by the introduction of a polar group (COOH) in the ACV molecule. Similarly, valacyclovir, as the first generation of nucleoside antiviral drug, was obtained from the chemical modification of ACV, which increased the water solubility of ACV [25]. Compared with the original drug, the oral bioavailability of valacyclovir was greatly improved. The fluorescent tricyclic analogues of acyclovir 6-(4-MeOPh)-TACV 8 showed similar antiherpetic potency as the parent compounds, but improved properties [26]. In addition, the modification of ACV can also improve its antiviral properties. With the help of lipid/calcium/phosphate nanoparticles (LCPNPs), monophosphorylation modification of ACV can successfully modify an anti-herpes simplex virus, thymidine kinase (HSV-TK) (HSV-TK) HSV-TK-dependent antiviral drug, into an anti-tumor drug [27]. ACV revealed preferential binding to Pd (II) through the N(7) position of the guanine nucleobase. The antiviral efficacy of the derived Pd (II) complex was assayed against HSV-1 strains and outperformed the antiviral activity against HSV-1 of ACV [28]. These modifications greatly improve the production of various dosage forms of ACV, providing more possibilities in treatments for human health.

### 3.3. The Dosage Forms

No drugs can be directly applied to patients. They must be made into drug dosage forms with certain shapes and properties before being applied in clinical practice so as to adequately achieve the drug’s effect, reduce toxic and side effects, and facilitate the drug’s use and preservation. Therefore, ACV is prepared in various dosage forms to serve different clinical needs, which can reduce the frequency of administration, increase bioavailability, maintain effective blood concentration, and prolong the duration of the drug effect. Currently, many available dosage forms are produced; they are briefly summarized in Table 2. In order to meet more clinical needs, many new dosage forms are being developed. A brief overview of these new dosage forms are as follows.

#### 3.3.1. Spray Formed In Situ

Topical application of ACV is normally done via ordinary emulsion and gel. The treatment is required to be applied with finger gloves or leather gloves, usually 5–6 times a day, 7–14 days a cycle. ACV can also be made into a spray, formed in situ with more uniform coating and more adjustable dosage. Polymer material quickly forms a slow-release film in situ after the spray contacts skin, evaporating the solvent, so that ACV is slowly released, topically. This achieves the therapeutic effect. Compared with ordinary spray, the retention of the drug at the target site can be prolonged; the efficacy can be improved; and the number of times the drug must be administered can be reduced. Moreover, a spray of ACV formed in situ does not contaminate clothing such that would cause the loss of liquid medicine. More importantly, a spray of ACV formed in situ is able to reduce the risk of infecting other parts of the body. Wu et al. [29] invented a method to prepare a spray formed in situ and secured a national invention patent. This patent of an in situ gel demonstrated a mechanism based on temperature sensitivity. Therefore, the applicable range can be expanded from eye administration to external use, such as skin and other places. A topical treatment of herpes simplex and herpes zoster infection is effective, which can meet a broad market demand.

#### 3.3.2. Gastric Retention Dosage Form

The bioavailability of ACV is only about 15–30% by oral absorption [30]. On the other hand, ACV has a short half-life of about 2.5 h, so it is required to take 200 mg orally 5 times a day for 10 days in the treatment of herpes, genital herpes and mucosal simplex for patients with immunodeficiency. This is very inconvenient. It was reported that ACV can have significantly increased absorption when slow administration is achieved, or by increasing its exposure time in the gastrointestinal tract [31]. There is a patent for the preparation of ACV gastric retention dosage form [32], which greatly improves its bioavailability. In addition, Ruiz-Caro et al. [33] developed mucoadhesive chitosan (CS) and hydroxypropyl-methylcellulose or hydroxypropylmethylcelluloseor (HPMC) tablets for gastric drug delivery of ACV. The results showed that HPMC and CS, with good mucoadhesion behavior on gastric mucosa, could be suitable polymers for obtaining tablets meeting ACV controlled release profiles. The formulation was reasonable, the preparation process was simple, and all data released for the studied tablets fitted the Hopfenberg model. Furthermore, Liu et al. [34] prepared a novel gastric mucoadhesive, sustained release ACV-resinate microsphere. Ion-exchange resin was used for adhesive delivery. The results indicated that the bioavailability of this ACV-resinate microspheres, with a sustained-release profile with an increasing retention time in the stomach in vitro with a better absorption ratio, was higher than conventional ACV tablets.

#### 3.3.3. Vaginal Sustained-Release Agents

**Scheme 3:**
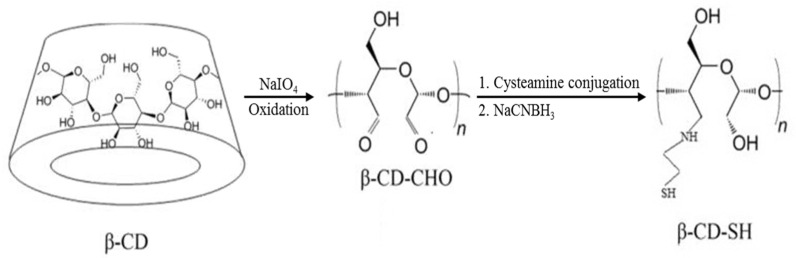
Schematic illustration of oxidation and subsequent cysteamine conjugation via reduction β-CD (slightly modified and reproduced with permission [37], copyright 2016, American Pharmacists Association).

Several investigations show that ACV is safe and effective for the treatment of primary or recurrent lesions from genital herpes [35]. The conventional dosage forms, such as creams, gels and tablets, may cause leakage, messiness and low residence time [36]. In order to solve these issues, vaginal sustained-release agents were studied by many researchers. Some have developed a liposomal vaginal delivery system for ACV [36]. However, on one hand, the percentage of drug encapsulation efficiency is low for liposomes. On the other hand, the fast drug release was developed in an acidic medium, inducing a low stability in simulated vaginal fluid. Recently, Ijaz et al. [37] synthesized a beta-cyclodextrin (β-CD) derivative with sulfhydry (β-CD-SH) to prepare inclusion complexes with ACV. Scheme 3 shows the process of synthesis of β-CD-SH. The inclusion complexes were applied in a porcine vagina, showing a good drug dissolution rate and vaginal adhesion. Moreover, Pacheco-Quito et al. [38] developed a vaginal tablet based on iodine–carrageenan and hydroxypropyl methyl cellulose for the controlled release of ACV. They evaluated the swelling, mucoadhesion and drug release in simulated vaginal fluid. The results showed high mucoadhesive capacity. This indicated that the formulation could stay in the vaginal area long enough to completely release the ACV.

## 4. Toxicology Studies of ACV

Thymidine kinase in normal human cells cannot phosphorylate ACV, so the toxicity of ACV is weak. Generally, the toxicity of ACV is related to the route of administration and dosage. A brief summary of the majority of patients’ experiences of side effects during medication is shown in Table 3. The subsequent recovery can be improved in the short term by adjusting the dosage or suspending the administration [39]. Numerous and extensive preclinical toxicity studies of ACV on animal and in vitro systems to detect the potential of acute, subchronic or chronic toxic manifestations, and the potential of teratogenesis, mutations or carcinogenesis have been reported. Multiple animal species were administered drugs in two or more routes, including intravenous and oral for each specific toxicological assessment.

### 4.1. Acute and Subchronic Manifestations of Toxicity

In 1982, Tucker [40] introduced the preclinical toxicological data for ACV. An acute toxicity test indicated that the oral median lethal dose (LD_50_) of ACV was greater than 10,000 mg/kg in mice and more than 20,000 mg/kg in rats, which has to do with the limited oral absorption of the drug in rodents. The LD_50_ of ACV obtained by intravenous and intraperitoneal routes in mouse and rat were also reported. Similarly, the LD_50_ values were relatively high, as shown in Table 4. According to British Pharmacopeia (BP2020), the medium lethal concentration (LC_50_) and LD_50_ of ACV are classified (Table 5). Although there are some available datasets on acute toxicity, the classification criteria have still not been met.

In 1983, a subchronic toxicity test for ACV was studied by Tucker [41]. After gavage of ACV at 50, 150 and 450 mg/kg/day for a month, the CD-1 mice showed no symptoms of toxicities. However, at the dose levels of 20, 40 and 80 mg/kg/day, rapid intravenous injection was applied on the rats once each day for 3 weeks; doing this, obstructive kidney disease was detected. In addition, Brigden [42] also summarized the acute toxicity of ACV and claimed its subchronic toxicity. Intravenous administration of 20 mg/kg/day or more can lead to obstructive crystal nephropathy in rats. Oral administration of 45 mg/kg and above daily, divided into three equal doses, may cause vomiting and diarrhea in dogs, with weight loss after the first week. These manifestations greatly contribute to a better understanding of the safe dosage when using ACV.

### 4.2. Chronic Manifestations of Toxicity

A chronic toxicity test of ACV was investigated in 1983 by Tucker [43] et al. The report showed that the dogs developed vomiting, diarrhea, ate less food, and weight loss within two weeks when ACV was fed at 150 mg/kg/day, while dogs that were fed at the dose of 45 mg/kg/day had fewer symptoms of gastrointestinal constipation. However, during the one-year trial, when these dose levels were subsequently reduced to 60 and 30 mg/kg/day, the symptoms of occasional and inconsistent vomiting and diarrhea appeared in the dogs. For the dogs treated with a mid and high dose of ACV, their paws were sore due to footpad erosion, and they had cracked, split and loosened nails during the 13th week of the study. Fortunately, the nails and footpads of dogs were normal at the dose of 15 mg/kg/day given throughout the study. It is not difficult to see that the usage of ACV has a good correlation with human health, showing that people must strictly follow the indications for selecting the usage and dosage of the drug, including the number of times of use and the route of administration to avoid excessive dosage and concentration.

### 4.3. Teratology and Reproductive Study

Many investigations have reported the applications of nucleoside analogues in human antiviral chemotherapy, which directly affect nucleic acid metabolism. The cytotoxicity produced has a profound effect on mammalian reproduction. In 1983, Moore et al. [44] found the potential for ACV to adversely affect the reproduction and development in laboratory animals. ACV was given subcutaneously to neonatal rats at the dose of levels of 5, 20 and 80 mg/kg/day for 19 consecutive days. It turned out that the effect of 20 mg/kg/day treatment on weight gain in neonatal rats was minimal, while that of the 80 mg/kg/day treatment was significantly reduced. However, minimal renal lesions were developed at the dose level of 80 mg/kg/day, without adverse signs of organ system development. Indeed, the doses of ACV can produce plasma concentrations well above the therapeutic level, thereby interfering with embryonic development in rats, and inducing typical gross structural abnormalities [45].

**Figure 2:**
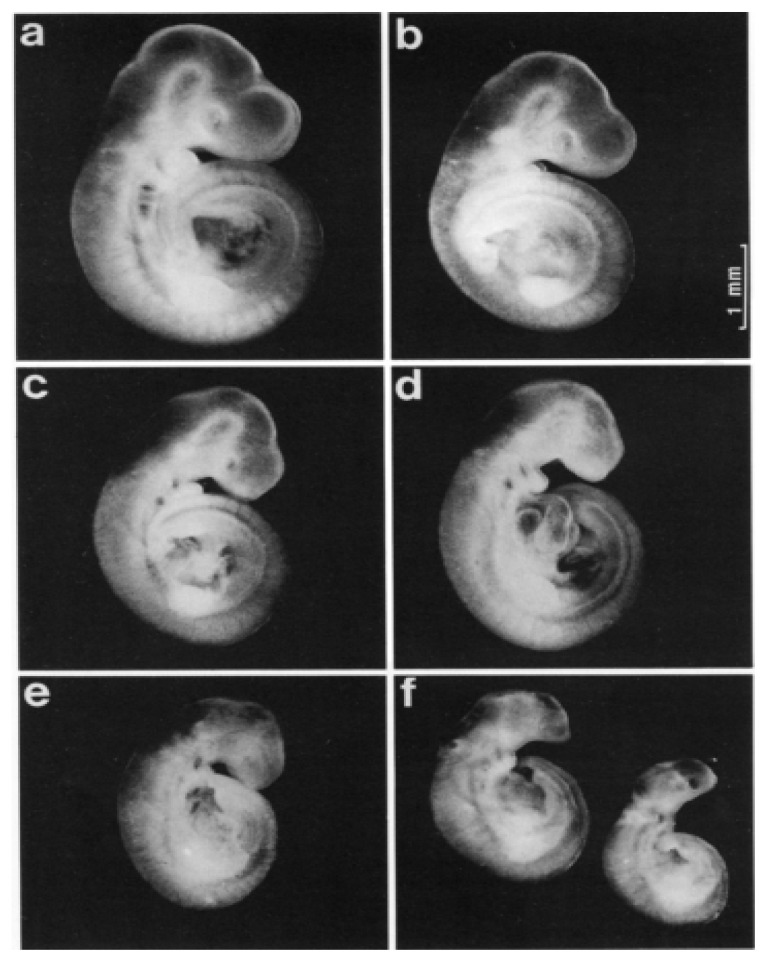
The 11.5-day-old rat embryos after maternal treatment with (**a**) 3 × 0.1 N NaOH (5 mL/kg) on day 10 of gestation (control); (**b**) 8 × ACV (50 mg/kg body wt) from day 9 (7:00) to Day 11 (12:00) of gestation; (**c**) 2 × ACV (100 mg/kg body wt) on day 10 (7:00 and 12:00) of gestation; (**d**) 1×ACV (200 mg/kg body wt) on day 10 (7:00) of gestation; (**e**) 3 × ACV (100 mg/kg body wt) on day 10 (7:00, 12:00, 17:00) of gestation; (**f**) 8 × ACV (100 mg/kg body wt) from day 9 (7:00) to Day 11 (12:00) of gestation (reproduced with permission [46], copyright 1991, Springer Nature).

A study by Klug et al. [46], aimed at exploring the prenatal toxicity of ACV in rats, found that ACV was shown to trigger teratogenic potency in vitro in the rat. Briefly, after eight injections of 50 mg/kg on days 9, 10 and 11, a reduction in the crown-rump length was observed, as shown in Figure 2b. However, this effect was more obvious after 100 mg/kg (Figure 2c). From Figure 2e, it can be clearly seen that embryonic abnormalities appeared, including an abnormal head shape and the width of the skull being decreased, resembling a beak-like visceral cranium, with this dose applied three times on day 10. When a single dose of 200 mg/kg was administered on day 10, the result shown in Figure 2d was similar to that shown in Figure 2e, but slightly more pronounced. To make matters worse, all the variables obtained changed dramatically after eight injections of 100 mg/kg on days 9, 10, and 11 (Figure 2f).

Unfortunately, the carcinogenic and mutagenic effects caused by ACV are still unclear, which requires that many researchers put in lot of effort, employing further advances in technology, to investigate.

### 4.4. Nephrotoxicity and Neurotoxicity

ACV is well tolerated in general, but in 1979, Selby et al. [47] firstly noticed impaired transient renal function after ACV administration. Subsequently, extensive studies were conducted on ACV, studying its nephrotoxicity and neurotoxicity [39,48,49,50,51,52,53]. ACV is mainly excreted through urine, after metabolism. Unfortunately, after rapid or excessive intravenous infusion, ACV becomes crystallized and forms precipitation in the renal tubules due to its low water solubility, which blocks the tubules, thus causing acute renal failure [49]. A recent study reported that the incidence of nephrotoxicity caused by ACV was 18–21% [54]. The renal function could be partially restored after ACV discontinuation. Bridgen et al. [55] actually found that fewer cases of renal impairment were developed after shifting from bolus to slow intravenous infusion over 1 h. Moreover, a case report from Meng et al. [52] found that acute renal failure was induced in a patient treated with facial neuritis by oral ACV for 8 days. Nevertheless, after the withdrawal of ACV, the renal function of the patient recovered completely, with continuing renal replacement therapy for 54 h, and supplemented by symptomatic treatment. ACV-induced acute renal failure has various manifestations, but the prognosis is still promising after active treatment.

In addition, side effects, such as drowsiness, insomnia, insanity, convulsions, hallucinations, tremors, mental disorders and coma, could develop, especially when the drug is administered intravenously or with susceptible factors (such as renal insufficiency). According to a recent report by Sadjadi [53], an 83-year-old African–American man received an intravenous dose of ACV, recommended by the manufacturer because of his shingles, a complication of end-stage kidney disease (ESRD) and chronic peritoneal dialysis (PD). Soon after taking it, he developed confusion, disorientation and visual hallucinations. Fortunately, after switching the treatment from PD to hemodialysis (HD), he restored.

## 5. Analytical Methods for Detection of ACV

Because of the excessive usage and large dosage of ACV, many other side effects and certain toxicity hazards will occur in animals. There are many analytical methods that can effectively quantify and detect ACV in commercial pharmaceutical formulations, human urine and serum. The application of existing analytical methods for the determination of ACV are vigorously discussed in the following.

### 5.1. Spectrophotometry

Spectrophotometry is a method for qualitative and quantitative analysis of a substance and continues to be the preferred means for routine analytical work. In fact, many colorless substances that do not absorb visible light can be changed into colored substances by chromogenic reactions so that they can be found by spectrophotometry, improving the sensitivity and selectivity of determination. Various kinds of chromogenic reactions exist, including complexation reaction, redox reaction, condensation reaction, etc. Therefore, there is no doubt that it is perfectly valid for applications to apply spectrophotometry in the assay of ACV.

**Figure 3:**
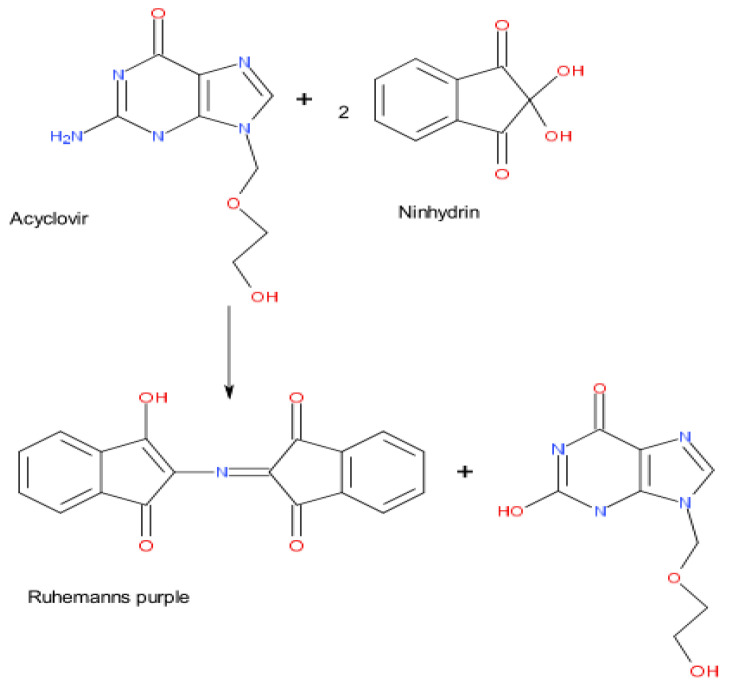
Proposed reaction pathway between ACV and ninhydrin–ascorbic acid (reproduced with permission [57], copyright 2015, Ukpe Ajima and Johnson Ogoda Onah).

According to the research by Sultan [56], ACV was found to experience an oxidative coupling reaction with 3-methylbenzothiazolin-2-one hydrazone (MBTH) in the presence of HCl and an the Fe(III) oxidant, obtaining a deep-green colored product. It is a very simple, clever and reliable spectrophotometric method for detecting ACV in pharmaceutical formulations with a limit of detection (LOD) of 1.06 μg/mL, and an analysis wavelength of 616 nm. Along with this, ACV can react with some other substances to produce new molecules, which can be tested by spectrophotometry. Ajima et al. [57] reported that the primary amino group of ACV was able to undergo condensation and a coupling reaction with ninhydrin–ascorbic acid in a citric acid buffer (pH 5), which created a purple-colored chromophore (Ruhemann’s purple) (Figure 3). At the maximum absorption wavelength of 540 nm, the concentration of ACV had a good linear relationship with the degree of color development, establishing a photometric method for the determination of ACV. After optimizing reaction conditions and interference tests, the method, which had high sensitivity (LOD: 0.3 μg/mL) and good selectivity, was suitable for application in the quality control of ACV in hospitals and laboratories. In addition to measuring ACV directly, indirect methods have caught many researchers’ attention. Kumar [58] developed an indirect method for ACV determination by spectrophotometry. In brief, excessive N-bromosuccinimide (NBS) was added to the acidic medium to oxidize ACV, and the subsequent residual amount of NBS was used to bleach the colors of a fixed amount of methyl orange. ACV was measured indirectly by the absorbance of methyl orange at 508 nm (LOD: 0.2 μg/mL). This method makes use of the relationship between ACV, NBS and methyl orange, which is a potential spectrophotometric method for ACV. In addition, the relevant literature on the detection of ACV by spectrophotometry [59,60,61] is summarized, and the main parameters are shown in Table 6.

### 5.2. High Performance Liquid Chromatography (HPLC)

**Figure 4:**
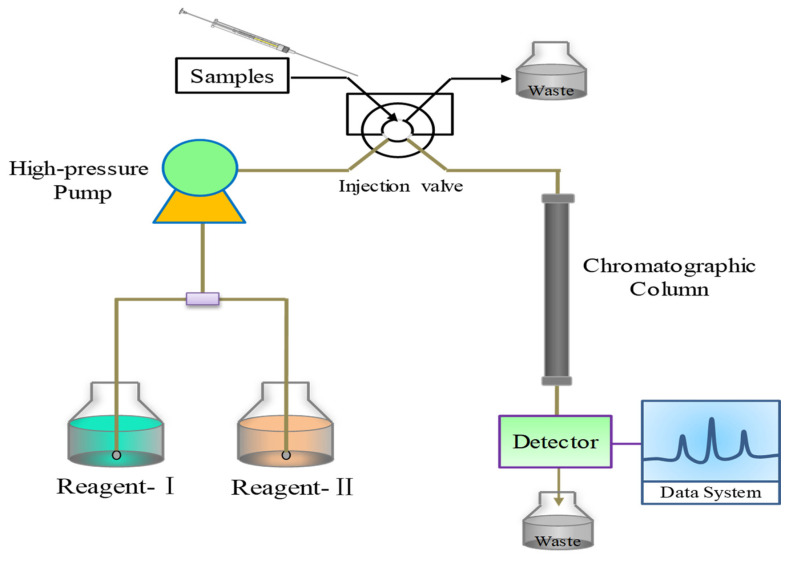
Schematic diagram of HPLC analysis system.

High performance liquid chromatography (HPLC), a chromatographic analysis method using liquid as the mobile phase, is one of the chromatography based techniques use to analyze ACV, generally, involving different detectors. Typically, ultraviolet-visible (UV-VIS) [62,63,64], fluorescence [65,66], photodiode array (PDA) [67] and diode-array detector (DAD) [68] are coupled with it. Octadecyl (C18) and monomer octyl (C8) stationary phase as the stationary phase are commonly used for efficient packing for reversed phase separations (Figure 4). At present, many pharmacopoeias, including Chinese Pharmacopoeia (CHP2015), United States Pharmacopeia (USP42), British Pharmacopeia (BP2020) European Pharmacopoeia (EP10), Japanese Pharmacopoeia (JP17), and South Korean Pharmacopoeia (KP 10), prescribe HPLC for the determination of ACV because this method is simple, fast and accurate.

Yet, the biggest challenge in determining ACV by HPLC is that the measured peaks are easily towed, and the retention time is relatively long. Therefore, the researchers studied effective detection conditions for assaying ACV in various environments. Silva et al. [69] validated and proposed that HPLC-UV be applied to detect ACV in vitreous humor with 0.02 mol/L acetic acid/methanol (95:5) as the mobile phase. Determination was carried out in a C_18_ column at 25 °C and UV detection at 254 nm. The recovery under those conditions was found to be in the range of 98.18–99.64%; the limit of quantitation (LOQ) was 0.16 μg/mL; and LOD was 0.048 μg/mL. The ACV attained a favorable and relatively short retention time and a symmetric chromatographic peak. It is also difficult for HPLC to effectively separate interferences in complex environments. Zendelovska et al. [70] employed a UV detection set at 255 nm on a reversed phase C_8_ column with 0.1% (*v*/*v*) triethylamine in water (pH 2.5) as mobile phase, to achieve the detection of ACV in plasma samples. Protein precipitation with 20% (*v*/*v*) perchloric acid was involved in the sample preparation, with a good separation of the ACV peak from peaks of endogenous interference compounds. This method was successfully adapted for use in the analysis of pharmacokinetic profiles of ACV tablets. The relevant literature on the detection of ACV by HPLC [71,72] was summarized, and the main parameters are shown in Table 7.

### 5.3. Liquid Chromatography/Tandem Mass Spectrometry (LC-MS/MS)

Liquid chromatography/tandem mass spectrometry (LC-MS/MS) is a chromatographic technique, which combines the advantages of the high separation performance of liquid chromatography and the high sensitivity and specificity of mass spectrometry. Similarly, its principles require certain types of mobile phase and other conditions to perform [73]. However, the difference is that the mass spectrometers are employed to overcome spectral interventions of PDA/UV-Vis detectors. Due to the sensitivity and specificity/selectivity of the technique, LC-MS/MS is usually chosen as a detector for highly accurate measurement. In terms of tandem mass spectrometry, quadrupole tandem mass spectrometry is predominantly used at present. It can perform MS1 and MS2 operations (in space). Kanneti et al. [74] produced an analysis method to simultaneously evaluate the quantification of ACV and valacyclovir with a mobile phase of 0.1% formic acid: methanol (30:70% *v*/*v*) by LC-MS/MS. The application in human plasma was validated successfully in the wide measurement range of 47–10,255 ng/mL for ACV, with a low LOQ (47.6 ng/mL). What is more, a new simple method with good sensitivity and accuracy by LC-MS/MS was applied in the determination of ACV and valacyclovir-HCl in tsetse flies from Sasanya et al. [75]. MgSO_4_ as well as MSPDC_18_ material were used as the solid phase, and the isocratic mobile phase consisted of methanol:acetonitrile:water (60:30:10, *v*/*v*/*v*) plus formic acid (0.1%). The overall accuracy of the method was 95% for ACV with the range of calibration of 0.45–4.5 μg/g. The LOQ for ACV was found to be 10.2 μg/g, and the recovery was found to be above 80%.

Unfortunately, those methods with quadrupole MS instruments suffered from high background signals since the endogenous peaks were eluted during the chromatographic separation. As technology progresses, LC-MS/MS are modified and improved. High-resolution mass spectrometry (HRMS) can not only accurately measure the mass of ions, but also determine their elemental (and isotopic) composition accurately. Ultra-high-performance liquid chromatography-high resolution tandem mass spectrometry (UHPLC-HR MS/MS) was used for introduced into the determination of ACV in human dermal interstitial fluid and serum form, as reported by Schimek et al. [76]. They indicated that the concentration range was from 0.1 ng/mL to 25 ng/mL with a sample volume of only 20 μL and validated the successful application with short-term and long-term sample stability data and the analysis of 5000 clinical trial samples. Furthermore, as the second mass analyzer, time of flight mass spectrometry (TOF) has become an important development direction, due to its high resolution, wide mass range, fast scanning and high sensitivity. Ultra performance liquid chromatography (UPLC) has the advantages of high analytical throughput, high sensitivity and large peak capacity by utilizing the theory and principles of HPLC and new technologies, such as rapid detection methods. Liquid chromatography-time of flight mass spectrometry (UPLC-TOF-MS) has also been used in the analysis of the degradation products of ACV and lidocaine in the samples [77]. The main parameters are shown in Table 8.

### 5.4. Electrochemical Analysis Technology

In recent years, the application of electrochemical analysis technology in pharmaceutical analysis has become much more widespread. It involves drug quality control, drug metabolism research, toxic substances detection and many other fields. It is inseparable from the advent and development of a large number of novel electrochemical sensors. Compared with the traditional analytical methods, the electrochemical analysis method has the advantages of simple operation, high sensitivity, low detection limit, quick response and easy in situ online detection. Various functional materials can be used as modifiers, such as graphene [78,79], nanometal particles [80], and nano-metaloxides [81,82,83,84], which can be introduced into electrochemical sensors. The comprehensive performance of the sensor can be further improved by amplifying the electrochemical signal and specifying the interaction between the functional material and the target molecule. ACV, with electrochemical activity, can produce a redox reaction whose mechanism on the electrode is validated by several studies [80,85,86], under the action of a certain electric field. Therefore, the quantitative relationship between analyte concentration and physical parameters can be established to detect ACV.

**Figure 5:**
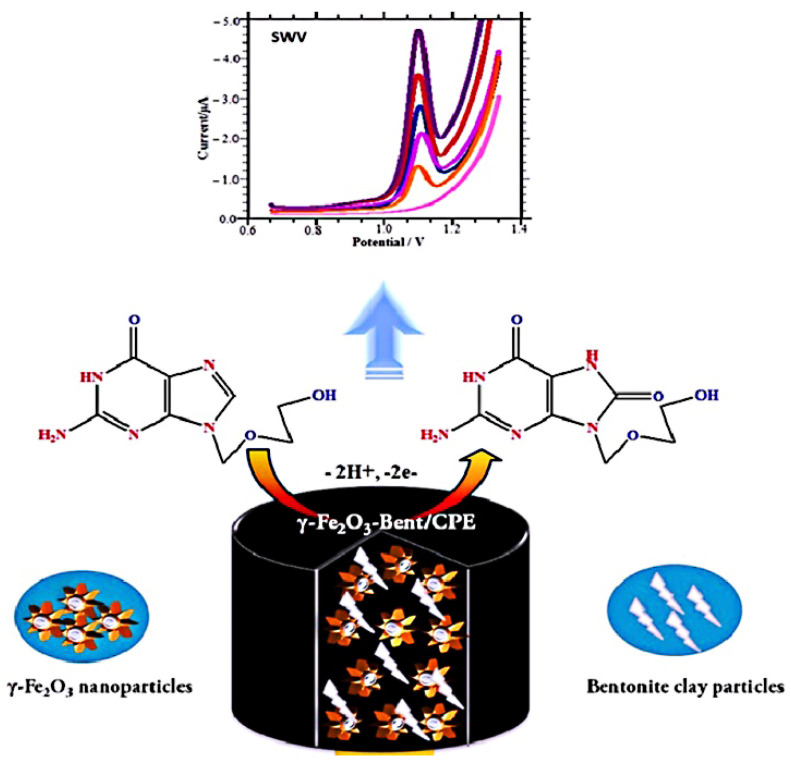
Schematic representation for theγ-Fe_2_O_3_-Bent/CPE detecting ACV (reproduced with permission [6], copyright 2020, Elsevier B.V.).

Shahrokhian et al. [87] prepared organic conjugated polymer polypyrrole (PPy) on the surface of MWCNT by electrochemical polymerization. PPY was coated on the surface of MWCNT with a porous film structure, which could enrich better ACV molecules. ACV tends to experience an irreversible oxidation reaction, involving two electrons and two protons on the electrode surface, producing 8-oxoacyclovir. Under the optimal experimental conditions, ACV has high redox activity such that we can quantitatively detect and rapidly evaluate ACV in tablets, injection and human plasma. In parallel, there are various modified materials and different kinds of electrodes that are employed. Recently, Shetti et al. [6] used composites that consisted of γ-Fe_2_O_3_ nanoparticles and bentonite clay particles to modify a bare carbon paste electrode (CPE) to prepare the γ-Fe_2_O_3_-Bent/CPE for ACV determination (Figure 5). They calculated the effective surface area of naked CPE and composite electrode, by using the Randles–Sevcik equation. The results showed that the effective surface area of the composite electrode was significantly larger than that of the naked CPE. Due to the large sensing area and excellent electrical conductivity, γ-Fe_2_O_3_-Bent produced a greater current intensity on the CPE surface during detection, resulting in a sensitive detection of ACV with an extremely low LOQ of 5.1 nM. In addition, other sensors, such as a pencil graphite electrode (PGE) [88], a glassy carbon electrode (GCE) electropolymerizated Eriochrome black T(PEBT) [89], a GCE modified with single-walled carbon nanotubes and nafion composite film (SWNT/Naf/GCE) [90], and so on, were also prepared for ACV assay; the major parameters are presented in Table 9.

### 5.5. Molecularly Imprinted Polymers (MIPs)

**Figure 6:**
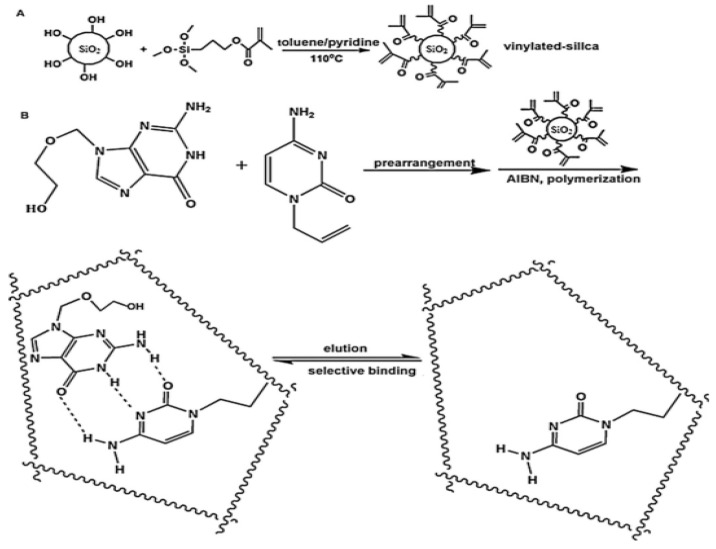
Preparation of MIMs, (**A**) synthesis of vinylated silica microspheres, (**B**) immobilization of MIPs on vinylated silica microspheres (reproduced with permission [97], copyright 2013, Elsevier B.V.).

Molecular imprinting is a prospective technology to synthesize molecularly imprinted polymers (MIPs) that have specific cavities matched with the target molecule. At present, with the advantages of low cost, simple preparation, being more environmentally friendly, specificity, affinity, high selectivity and having high stability for the target analyte, MIPs are widely used in chemical sensors, solid-phase extraction, artificial antibodies and other fields [96]. Typically, an unbalanced structure of MIPs will occur when local temperature changes during exothermic polymerization, which can further cause the final polymer morphology to be uneven on the length scale. Resourcefully, Wu et al. [97] engineered a novel approach to assemble homogeneous MIPs by mimicking multiple hydrogen bonds between nucleotide bases and grafting on silica supports with ACV as a template molecule, which obtained the balanced structure of MIPs. Briefly, the silica microsphere surface was activated to vinylated silica microspheres via the process shown in Figure 6A. Subsequently, the vinylated silica microspheres were subject to polymerization with ACV and allyl-cytosine under the initiator of 2, 2′-azobisisobutyronitrile (AIBN). After cleaning the solid polymers and eluting the ACV from inside, molecularly imprinted microspheres (MIMs) were finally prepared (Figure 6B). MIMs, as a specific solid phase extraction coupling with HPLC, were successfully applied in capturing and detecting ACV from serum samples. The LOD and mean recovery were 1.8 ng/mL and 95.6%, respectively.

In addition to the above construction methods, using MAA as the functional monomer, EGDMA as the crosslinking agent and AIBN as the initiator is a typical method to construct a molecular imprint. A study by Yan et al. [98] suggested that miniaturized molecularly imprinted solid-phase extraction (mini-MISPE) coupled with HPLC can be applied to the assay of ACV in urine. Hybrid molecularly imprinted polymers (HMIPs) were synthesized by polymerizing new hybrid monomers (3-aminopropyltriethoxysilane-methacrylic acid, APTES-MAA), theophyllines (as dummy templates), ethylene glycol dimethacrylate (EGDMA, as a cross-linking agent) and tetraethoxysilane (TEOS) under the initiator of AIBN. Finally, the HMIPs were filled into a milliliter tapered plastic centrifuge tube for solid-phase extraction. Then, a selective screening of ACV was carried out by HPLC. The results indicated a good linear calibration (R^2^ = 0.9994) and an acceptable range of mean recovery (91.6–103%) for ACV. Alongside, Han et al. [99] prepared the molecularly imprinted matrix solid-phase dispersion (MI-MSPD) by using templates of theophyllines, functional monomers of MAA, a cross-linking agent of EGDMA and an initiator of AIBN. A simple, rapid and selective method, which applied MI-MSPD coupled with HPLC (MI-MSPD-HPLC), was proposed for analyzing ACV in creatural tissues.

### 5.6. Flow Injection–Chemiluminescence (FI-CL)

**Figure 7:**
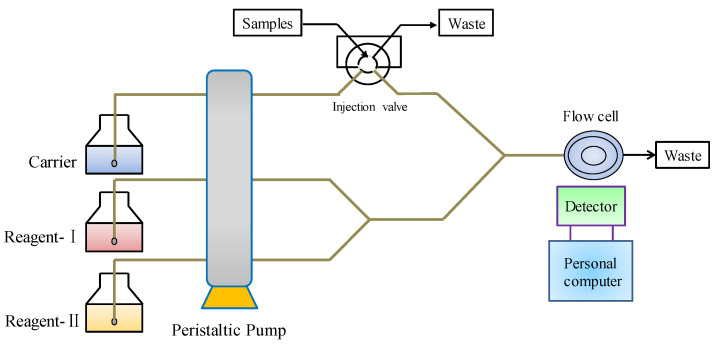
Schematic diagram of FI–CL analysis system.

Flow injection–chemiluminescence (FI-CL) is a powerful and effective analysis technique, combining chemiluminescence (LC) analysis with a flow injection technique [100]. The schematic diagram of FI-CL is shown in Figure 7. Because of its advantages of high sensitivity, wide linear range, simple instruments and convenient operation, it is widely used in environmental monitoring, drug analysis, food analysis and so forth [101].

**Figure 8:**
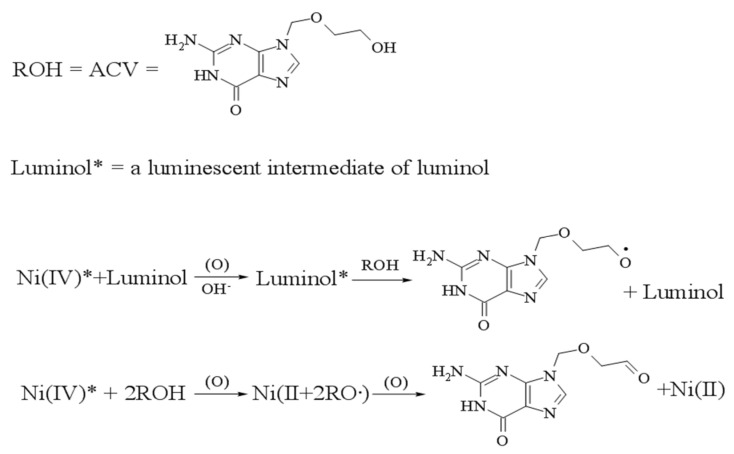
Chemistry mechanism for the ACV detection in Reference [103].

Using alkaline as medium, ACV can significantly inhibit Ni (IV) complexes-luminol LC systems, and there is a linear relationship between the inhibition extent and the concentration of ACV within a certain range. Hereby, Li et al. [102] established a Ni (IV) complexes–luminol LC system for the detection of ACV by FI-CL. As Figure 8 shows, the Ni (IV) complexes can react with luminol to produce a luminescent intermediate of luminol. But Ni (IV) complexes could not react with ACV. However, the luminescent intermediates transfer part of the free radicals to ACV, thereby reducing the free radicals of luminol, then reducing the amount of laminator. Finally, the luminous intensity of the system is reduced. Therefore, luminol solution and Ni (IV) complexes were used as the reagent-Ι and reagent-II, respectively. Driven by the borax buffer solution (carrier), the injected ACV met and reacted with the intermediate. The results showed that there was a good linear relationship with CL intensity in the range of 50–1200 μg/L. It was found that the LOD was 0.03 mg/L, with a satisfactory stability.

Additionally, ACV at low concentration might enhance the CL of the luminol–H_2_O_2_ system in an alkaline medium. It could be directly proportional to the CL intensity of the luminol–H_2_O_2_ system, which allows the determination of ACV in the linear range 0.09–3 μmol/L. The method showed a low LOD for ACV (77.1 nmol/L), as well as a low relative standard deviation (RSD) of 0.43% (11 times in parallel at the concentration of 1.00 μmol/L). It was successfully applied to the determination of ACV.

Elsewhere, Long and Chen [103] found that ACV could react with potassium permanganate to generate CL in the presence of formaldehyde in acidic solution. Based on that, they created a new method for determination of ACV by FI-CL, which showed high selectivity, wide linearity (0.2–80 mg/L) and low LOD (0.06 mg/L).

## 6. Current Research and Future Challenges of ACV

### 6.1. Current Research

ACV has problems, such as low bioavailability and development of virus resistance. How to improve the effective access to the target site and the antiviral ability of ACV is the focus of current research. Many researchers have tried a major strategy of water-triggered labile ACV precursors to achieve such an aim. Zhou et al. [104] used acid anhydride acylation to synthesize three ACV precursor drugs, including acetate (ACV-Ace), butyrate (ACV-But) and hexanoate (ACV-Hex), to prepare a supersaturated system of lipophilic ACV precursor drug, which increased the skin bioavailability of ACV. Ljiljana et al. [105] prepared six self-dispersion systems (SD1-SD6), whereby ACV was packed into a semi-solid formulation consisting of medium chain triglycerides. Semisolid self-microemulsifying drug delivery systems (SMEDDSs) were developed as carriers for rigid hydroxypropylmethylcellulose (HPMC) for oral ACV.

Meanwhile, many analogues of ACV nucleosides were synthesized and corresponding formulations were fabricated. For examples, valacyclovir [106], ganciclovir [107], and valganciclovir [108] are now available as anti-herpesviral drugs. Sahu et al. [109] synthesized a series of acyclic selenopurine nucleosides based on the principle of biological substitution between oxygen and selenium, and among the compounds tested, selenium–acyclovir exhibited the strongest antiherpes simplex virus ability and activity. Komazin et al. [110] investigated the involvement of HHV-6 U69 protein kinase in their mechanism of action. The phosphorylation of the dihydroxymethyl analogue cyclopropavir and monohydroxymethyl nucleosides with either a 6-ether moiety (MBX 2168) or a 6-thioether moiety (MBX 1616) with purified U69 was examined. All three compounds were substrates of this viral kinase. These studies provide more new types of structures for the development of ACV-like antiviral drugs that can more effectively address the shortcomings of ACV, while improving antiviral capabilities and bringing a more open perspective for disease treatment.

### 6.2. Future Challenges of ACV

Because of the complexity of diseases and the diversity of drug properties, many researchers are contributing to the development of various ACV delivery systems to meet different needs. Oral instant tablets, which can be taken without water, are of great convenience to patients [111]. The side effects caused by ACV should also be avoided, which are related to the dosage and the speed of administration. Therefore, the development of long-term sustained-release injection, with slow release for one month or three months at a time, can not only overcome the pain of daily injection, but also reduce the toxic and side effects by maintaining a stable blood concentration. Furthermore, ACV has a special affinity for viruses, but is less toxic to mammalian host cells [112]. Although carcinogenesis was reported in vitro, no evidence of carcinogenesis was found in animal experiments. Some animal studies have shown that high concentrations of the drug can cause mutations, though there is no evidence of chromosomal changes. The carcinogenic and mutagenic effects of ACV are unclear. Furthermore, great efforts are being made to reduce, refine, and replace the use of animal models in various fields of biomedical research.

Given this challenge, organ-on-a-chip, which are not simulators using silicon electronic chips to simulate human organs, but biochips containing real, living human cells, are widely expected to be used in future drug toxicology and drug discovery [113,114,115]. Lee and Sung’s [116] recent research aimed at investigating drug toxicology and effects by reconstructing physiological microenvironments in vitro. As a recent example, a bioanalytical platform was developed by microfluidic culture of human hepatocytes, coupled with NMR spectroscopy to monitor the metabolic responses of hepatocytes to flutamide (an anticancer drug) and hydroxyflutamide (flutamide’s active metabolite), both of which are hepatotoxic [117]. By quantitative analysis and plotting of metabolism and mitochondrial activity, the metabolic characteristics of toxic drug reactions in the model were validated. Moreover, the metabolic pathways involved in the induction of hepatotoxicity were delineated, between those due to flutamide and hydroxyflutamide. The organ-on-chip approach was employed in this study; it is conducive to the reduction of biological noise inherent to the in vivo metabolomics model and reveals a potential source of hepatotoxicity-specific biomarkers. Organ-on-chip is an amazing technology being utilized not only to evaluate human-related drug reactions with expected toxicity at different levels of biological complexity, such as subcellular, cellular, tissue, and organ levels, but also to assess unexpected off-target toxicity [118]. Promisingly, the unknown role of ACV in the human body could be explored with the help of organ-on-a-chip technology in the future, due to its promising prospects. A new understanding of ACV may be proposed soon, which can be expected to have great research significance.

In terms of computational methods in ACV discovery, drug research and development based on wet experiments is an expensive, time-consuming and challenging process. As an effective virtual shortcut, computer-aided drug design helps to shorten the long process and reduce the cost of drug design. Now, computer-aided drug design has become an indispensable tool in drug research and development [109,110,111,112,113,114,115,116,117,118,119,120]. More importantly, increasing the knowledge of diverse chemical structures, as well as the boosting of computer power, makes it possible to discover new clues for treatment using many available pharmaceutical drugs approved by the FDA that have reached the market. Computation-based drug discovery methods can be roughly classified into structure-based drug design, ligand-based drug discovery, network-based drug repositioning, and machine learning–based drug repositioning [121].

Structure-based drug design methods assume that the 3D structures of a drug target are known and design a computation method to find new treatment clues of drugs based on the target structure. Structure-based drug discovery methods contain molecular docking algorithms and de novo ligand (such as antagonists, inhibitors of a target) exploitation. Popular structure-based prediction tools include homology modeling tools (such as Swiss model [122], Phyre2 [123], and RaptorX [124]), fold recognition tools (such as MUSTER [125] and I-TASSER [126]), and de novo modeling tools (such as QUARK [127] and EVfold [128]). Ligand-based drug discovery methods consider the conditions where drug target structures are unknown and thus, design multiple drug discovery models, based on ligand structures. These methods contain pharmacophore modeling approaches [129], molecular similarity approaches [130] and quantitative structure–activity relationship modeling approaches [131]. Network-based drug discovery methods use network propagation algorithms and diverse biological networks, including a drug–target interaction network, drug–disease interaction network and drug–drug interaction to reposition existing FDA-approved drugs and find their new treatment potential. Machine learning–based drug repositioning obtains feature vectors of FDA-approved drugs based on drug biological information and develops effective classifiers to reposition the available drugs.

The above four types of drug discovery methods can be effectively applied to boost the drug discovery process; they are widely used by pharmaceutical companies and academia. Now, the methods are one valuable part of the drug discovery pipeline and demonstrate great promise. Therefore, we can fully utilize the four types of methods to find related therapeutic clues for ACV. However, structure-based methods require that the structures of receptors are known when finding target candidates of ACV. Therefore, the combined ACV methods integrating structure-based and ligand-based methods can amplify their advantages and improve this identification.

Network-based drug discovery methods [132,133] use drug similarity in the biological networks, the similarity of other biological entities, and network algorithms. Therefore, we can find new uses of ACV, based on network-based drug discovery methods. However, network-based methods could not be applied to drug function prediction for orphan drugs. Thus, the type of method may be unavailable when there is no information associated with ACV.

Machine learning–based drug discovery methods [134,135,136] effectively take advantage of the optimal performance of machine learning and obtain better drug prediction performance. This type of method selects the biological features of drugs and other associated entities from the existing databases and develops a classification model to classify drug–target interactions, drug–disease interactions, and drug–drug interactions. We can find possible targets, diseases, and other drugs interacting with ACV based on machine learning models. Generally, machine learning–based methods, especially deep learning models, can obtain the best prediction performance for finding new biological functions of ACV.

## 7. Conclusions

ACV is one of the most common and broad-spectrum antiviral drugs. It is widely and extensively used against herpes simplex virus (HSV), herpes simplex, and varicella-zoster virus. This article reviews the synthesis, modification, and detection methods of ACV. Although synthetic methods of ACV are relatively mature for now, with the development of drug analysis theory and application technology, new methods have emerged. At present, there are a variety of methods for determining ACV, such as spectrophotometry, chromatography, and MIPs. In recent years, electrochemical analysis methods based on modified working electrodes have attracted widespread attention in the field of pharmaceutical analysis, due to their simple operation, rapid response, and high sensitivity as well as selectivity, low cost and good reproducibility. Finally, in response to the future challenge of reducing, refining and replacing the use of animal models, it is concluded that machine learning approaches, especially deep learning models, have made significant contribution to the efforts to find new biological functions of ACV.

## Figures and Tables

**Table 1:** Various starting materials for ACV synthesis.

	Starting Materials		Synthesizing Process	The Routes in Scheme 2	References
(1)	5-aminoimidazole-4-carboxamide		Three steps: alkylation, condensation, ring closure	(a)	[11,12]
(2)	Guanine and its derivatives	Guanosine	Three steps: acylation, condensation, azmmonolysis	(b)	[13]
		Guanine	Three steps: acylation, condensation, ammonolysis	(c)	[14,15]
			Three steps: silanization, condensation, hydrolysis	(d)	[16]
			One step: the condensation and deprotection of the acyl protecting group and nucleophilic displacement of the halogen atoms	(e)	[17]
(3)	other purine derivatives	2, 6-dichloropurine	Condensation, ammoniation and hydrolysis	(f)	[18]
		2-chloro-6-iodopurine	Condensation, ammoniation and hydrolysis	(g)	[19]
		6-iodopurine	Alkylation, chlorination, iodization, ammoniation	(h)	[20]
		2-amino-6-chloropurine	Silanization, condensation and hydrolysis	(i)	[21]

**Table 2:** Currently available dosage forms.

Dosage Forms	Products
Solid Preparation	ACV tablets, ACV dispersible tablets, ACV sustained-release tablets, ACV chewing tablets, ACV capsules, ACV granules
Semi-Solid Preparation	ACV eye cream, ACV cream, ACV ointment, ACV gel
Liquid Preparation	ACV for injection, ACV oral suspension, ACV eye drops

**Table 3:** Side effects induced by ACV.

Human System	Chief Clinical Manifestations
Urinary System	No urine, oliguria, low back pain, etc.
Nervous System	Headache, sudden neurological abnormalities, etc.
Cardiovascular System	Low blood pressure, palpitations, etc.
Digestive System	Diarrhea, nausea, vomiting, etc.
Allergic Reactions	Itchy skin, urticarial, anaphylactic shock, etc.

**Table 4:** Acute toxicity of ACV (“N.A”: No data applicable or available).

	Mouse		Rat	
	Male	Female	Male	Female
LD_50_ by intravenous route (mg/kg)	405	N.A	>600	N.A
LD_50_ by intraperitoneal route (mg/kg)	1454	999	1305	1210

**Table 5:** LD/LC_50_ values relevant classification (“N.A”: No data applicable or available).

	LD_50_	LC_50_
Oral	20,000 mg/kg (rat)	N.A
Inhalative	Intraperitoneal: 860 mg/kg (rat) Intravenous: 750 mg/kg (rat)	>15.1 mg/L (rat)

**Table 6:** Various spectrophotometry methods for the detection of ACV.

Methods	Operating Wave Length(nm)	Linear Range (μg/mL)	LOD (μg/mL)	Samples	Recovery%	References
UV/Vis	616	20–200	1.06	dosage forms of ACV	98.6–99.7	[56]
UV/Vis	540	<30	0.2	dosage forms of ACV	96.9–102.0	[57]
UV/Vis	508	1–5, 5–10	0.3	ACV and valacyclovir in commercial tablet	99.26 ± 0.52, 99.47 ± 0.96	[58]
^a^ DS	295.2	1.25–40.0	0.08	^b^ ACV present in PACA nanoparticles	99.3–101.2	[59]
UV/Vis	404	1.81–9.06	0.024	ACV in bulk and dosage forms	97.0–100.0	[60]
UV/Vis	252	1600–2400	0.030	ACV in bulk and pharmaceutical Dosage form	99.72	[61]

^a^ Derivative spectrophotometry; ^b^ Poly (n-butylcyanoacrylate).

**Table 7:** Various HPLC methods for the detection of ACV (“N.A”: No data applicable or available).

Methods	Operating Wavelength (nm)	Linear Range (μg/mL)	LOD (μg/mL)	Samples	Recovery%	References
HPLC-VIS	254	0.02–4	0.01	plasma samples	94.4–104.3	[62]
HPLC-VIS	254	0.010–2.560	0.01	ACV in human serum	90 ± 8%	[63]
HPLC-VIS	254	0.25–100	2.5	Plasma and amniotic fluid samples	>80%	[64]
HPLC-FLD	260	100–20,000	33	Plasma from pediatric oncology patients	101%	[65]
HPLC-FLD, LC-HESI-MS/MS	N.A	0.1–1.5	0.05, 0.002	equine plasma and body fluids	98.8–99.3, 95.4–99.9	[66]
HPLC-PDA	350	50–500	N.A	ACV (ACV Hospira^®^)	N.A	[67]
HPLC-DAD	N.A	100–1000	20	ACV raw material	99.2–101.6	[68]
HPLC-UV	254	35–70	0.048	ACV in the vitreous humor	98.18–99.64	[69]
HPLC-UV	255	0.1–5.0	N.A	ACV in plasma samples	91.37–98.98	[70]
HPLC-UV	254	0.001–5	0.0I	a pharmacokinetic linearity study in dogs.	88.2–92.9	[71]
^a^ YMC-Triart C18	252.0	8–12	0.27	Aciclovir Cream	99. 6–100.6	[72]

^a^ A reverse phase chromatographic.

**Table 8:** Various LC-MS/MS methods for the detection of ACV.

Methods	Linear Range (μg/mL)	LOD (μg/mL)	Samples	Recovery%	References
^a^ UPLC-MS	50–500	50	9-carboxymethoxymethylguanosine in human serum	92.2–114.2	[70]
HPLC-FLD, LC-HESI-MS/MS	0.1–1.5	0.05, 0.002	equine plasma and body fluids	98.8–99.3, 95.4–99.9	[63]
LC-MS-MS	0.047–10.255, 0.005–1.075	0.047, 0.005	ACV and valacyclovir in human plasma.	96.0–106.3, 99.2–105.5	[71]
^b^ HPLC- MS,	0.45–4.5	0.0625, 0.2	valacyclovir-HCl and ACV in tsetse flies	92%, 95%	[72]
^c^ UHPLC-HR MS/MS	0.0001–0.025	0.00001	ACV in dermal samples	82 ± 5%	[73]
^d^ UPLC-TOF-MS	5–500, 10–200	5, 10	ACV and lidocaine in topical formulations	92.8 ± 0.7, 91.3 ± 3.2	[74]

^a^ Ultra-high-performance liquid chromatography coupled with mass spectrometry method; ^b^ Liquid chromatography combined with heated electrospray ionization tandem mass spectrometry; ^c^ Ultra-high-performance liquid chromatography-high-resolution tandem mass spectrometry method; ^d^ Ultra performance liquid chromatography-time of flight mass spectrometry.

**Table 9:** Various electrochemical sensors for the detection of ACV (“N.A”: No data applicable or available).

Electrochemical Sensors	Technique	Linear Range (μM)	LOD (μM)	Samples	Recovery%	References
Copper nanoparticle/CPE	Amperometry	27–521	2.64	Tablets	96.5–106	[80]
Fullerene-C_60_/GCE	^g^ DPV	0.090–6.0	0.0148	Urine; Human serum	98.12–99.43	[85]
^a^ MBZ/TMHPP Cu(II)/GE	^h^ SWV	0.01–1000	0.01	Tablets; Urine	100–102	[86]
PGE	DPV	1–100	0.30	Tablets	N.A	[88]
PEBT/GCE	DPV	0.03–1.5	0.012	Tablets; Human serum	98-102	[89]
SWNT/Naf/GCE	SWV	0.01–30	0.0018	Tablets	96.0–102.7	[90]
^b^ MWNTs-DHP/GCE	LSV	0.080–10	0.030	Tablets	102–105	[91]
^c^ UTGE;GCE	DPV	4–70; 2.0–100	1.0; 0.35	Human urine	104; 99	[92]
^d^ PVP/CPE	DPV	0.01–0.75	0.0025	Injection; Tablets	N.A	[93]
^e^ ZnO/GCE	SWV	1–20	0.0423	Tablets; Urine	91.3–97.5	[94]
^f^ PS:β-CD IC/Y_2_O_3_/GCE	DPV	0.01–118; 148–918	0.02	Urine; Tablets	88–99.3	[95]

^a^ A gold electrode (GE) modified with a self-assembled monolayer of 2-mercaptobenzothiazol (MBZ) and [5,10,15,20] tetrakis(3-methoxy-4-hydroxyphenyl)porphyrinato]-copper(II)(TMHPP Cu(II)); ^b^ A multi-wall carbon nanotubes (MWNTs)-dihexadecyl hydrogen phosphate (DHP) film-coated GCE; ^c^ Ultra-trace graphite electrode; ^d^ Polyvinylpyrrolidone (PVP) modified CPE; ^e^ ZnO nanoparticles modified GCE; ^f^ Polystyrene:β-cyclodextrin inclusion complex-supported yttrium oxide (Y_2_O_3_)-modified GCE; ^g^ Differential pulse voltammetry; ^h^ Square wave voltammetry; Linear sweep voltammetry.
